# The Pubovesical Complex–Sparing Laparoscopic Radical Prostatectomy Improves Early Urinary Continence Without Compromising Oncologic Safety: A Prospective, Randomized, and Double‐Blinded Clinical Trial

**DOI:** 10.1002/pros.70106

**Published:** 2025-12-07

**Authors:** Rafael Batista Rebouças, Matheus da Costa Souto, Ana Luiza Jácome Franca Campos, Rodrigo Campos Monteiro, Alcides de Assis Lira Neto, Cesar Araújo Britto, Patrícia Candido, Poliana Romão, Alberto Azoubel Antunes, William C. Nahas, Sabrina T. Reis, Carlo Camargo Passerotti

**Affiliations:** ^1^ Universidade de João Pessoa, UNIPE João Pessoa Paraíba Brazil; ^2^ Hospital São Vicente de Paulo João Pessoa Paraíba Brazil; ^3^ Hospital Universitário Lauro Wanderley Universidade Federal da Paraíba João Pessoa Paraíba Brazil; ^4^ Hospital Universitário Onofre Lopes Universidade Federal do Rio Grande de Norte Natal Rio Grande do Norte Brazil; ^5^ Laboratory of Medical Investigation (LIM55), Urology Department Faculdade de Medicina da Universidade de São Paulo São Paulo Brazil; ^6^ Moriah Institute of Science and Education Hospital Moriah São Paulo Brazil; ^7^ Division of Urology University of São Paulo Medical School São Paulo Brazil; ^8^ Center for Robotic Surgery Hospital Alemão Oswaldo Cruz São Paulo Brazil

**Keywords:** laparoscopic radical prostatectomy, minimally invasive surgery, prostate cancer, pubovesical complex, urinary incontinence

## Abstract

**Background:**

Post‐prostatectomy urinary incontinence significantly impacts quality of life. Techniques that preserve periprostatic structures have shown promise in promoting earlier continence recovery, particularly with robotic‐assisted surgery. The study aimed to evaluate the effect of pubovesical complex (PVC) preservation on urinary continence recovery in patients undergoing laparoscopic radical prostatectomy (LRP).

**Methods:**

In this randomized, blinded, prospective clinical trial, 72 patients with localized prostate cancer were assigned to standard LRP or LRP with PVC preservation. The primary endpoint was urinary continence recovery, defined as complete absence of leakage or pad use, assessed at 24 h, 15 days, 1, 3, and 6 months post‐catheter removal. Secondary endpoints included operative time, blood loss, complications, and oncologic outcomes.

**Results:**

At 6 months, continence was significantly higher in the PVC group (82.4% vs. 57.6%; *p* = 0.027). Earlier timepoints showed improved, though not statistically significant, continence rates in the PVC group. Operative time (109 vs. 75 min; *p* < 0.001) and blood loss (365 vs. 247 ml; *p* = 0.010) were greater with PVC preservation. Complication and margin positivity rates were similar between groups.

**Conclusion:**

PVC preservation during LRP significantly improves urinary continence recovery without compromising oncologic safety. This accessible technique can be adopted in centers lacking robotic platforms, offering equitable benefits for patients in resource‐limited settings.

**Trial Registration:**

Brazilian Clinical Trials Registry (ReBEC), RBR‐7f25wsz.

## 1 Introduction

Surgical treatment of localized prostate cancer should aim not only oncological control but also optimal functional outcomes. The concept of *pentafecta*, introduced in 2011, defines an ideal surgical result as the combination of urinary continence and sexual function preservation, absence of biochemical recurrence, negative surgical margins, and no perioperative complications [1].

Although advancements in surgical techniques and the introduction of robotic platforms have aimed an enhancement of functional outcomes, urinary incontinence remains one of the most concerning postoperative complications for both patients and surgeons.

Radical prostatectomy techniques that preserve the anterior anatomical structures of the prostate have been associated with improved urinary continence outcomes, particularly regarding immediate or early recovery, as observed in the Retzius‐sparing approach [2].

The technique of preservation of the pubovesical complex (PVC), proposed in 2010, consists of a robotic approach in which the prostate and seminal vesicles are accessed laterally, allowing the en bloc removal of the specimen without transection or ligation of the PVC. The vesicourethral anastomosis is then performed bilaterally around the preserved complex. This non‐randomized series included 30 cases, reporting an immediate continence rate of 80%—the highest rate documented in the literature up to that point [3]. However, to date, there is no randomized controlled trial comparing the PVC—sparing with the standard techniques.

A laparoscopic technique capable of reproducing the functional outcomes of recent robotic preservation approaches could greatly enhance the quality of life of prostate cancer patients, especially in regions with limited access to robotic surgery. In 2018, we described for the first time in literature, the feasibility and safety of a PVC—sparing technique using a pure laparoscopic approach [4].

In the present article, we aimed to perform the first prospective, randomized study comparing the PVC—sparing technique with the standard radical prostatectomy using a pure laparoscopic approach, and its impact on the urinary continence rates.

## 2 Material and Methods

### 2.1 Study Design

This randomized, prospective, controlled, and blinded clinical trial included 72 patients with localized prostate cancer who underwent laparoscopic radical prostatectomy (LRP). Patients and outcome evaluators were blinded. As the surgeon could not be blinded, the study was conducted in a single‐blind design.

All procedures were performed by a single experienced surgeon with prior experience of more than 300 laparoscopic radical prostatectomies, including 10 cases using the PVC preservation technique.

Patients were divided into two groups: Group 1 underwent LRP with complete preservation of the PVC, while Group 2 underwent the standard laparoscopic technique, involving transection of the dorsal venous complex followed by selective ligation. All procedures were performed by a single experienced surgeon.

### 2.2 Patients

The sample size (*n* = 72) was calculated using a paired sample qualitative analysis formula, considering the primary endpoint of immediate continence. Randomization was performed using a computer‐generated random table.

Eligible participants were patients younger than 75, with low and intermediate‐risk PC, classified according to the National Comprehensive Cancer Network (NCCN) [5].

Exclusion criteria were preoperative urinary incontinence; prostate volume more than 80 ml; neoadjuvant cancer therapy; prior prostate, bladder, or urethral surgery; biopsy involving the anterior region of the prostate; vascular or neurological comorbidities; loss of follow‐up/data; failure to complete the randomized surgical procedure.

### 2.3 Data Collection

On preoperative evaluation, the following data were collected: age; body mass index (BMI); prostate volume; comorbidities, clinical stage; PSA; the number of fragments; Gleason/ISUP score; IIEF‐5 sexual potency questionnaire [6]; micturition symptoms score (IPSS) [7].

Operative data was collected: operative time, estimated blood loss, transfusion, complications, and data on preserving the neurovascular bundles.

The postoperative data collected were: length of hospitalization; time of bladder catheterization; postoperative complications measured according to the modified Clavien classification [8]; PSA (30 days post catheter removal, 3 and 6 months); IIIEF‐5 questionnaire (30 days post catheter removal, 3 and 6 months); urinary continence (defined as the complete absence of urinary leakage) assessed 24 h after catheter removal, 15 days, 30 days, 3 and 6 months; and pathological analysis data.

### 2.4 Statistical Analysis

Categorical variables were compared between groups using the chi‐square test or Fisher's test. Students' *t*‐tests or Mann‐Whitney tests were used to compare numerical variables for homogeneous or nonhomogeneous variables. In all analyzes, differences were considered significant when they obtained *p* < 0.05. All analyzes were performed using SPSS 22.0 software.

## 3 Results

**Figure 1:**
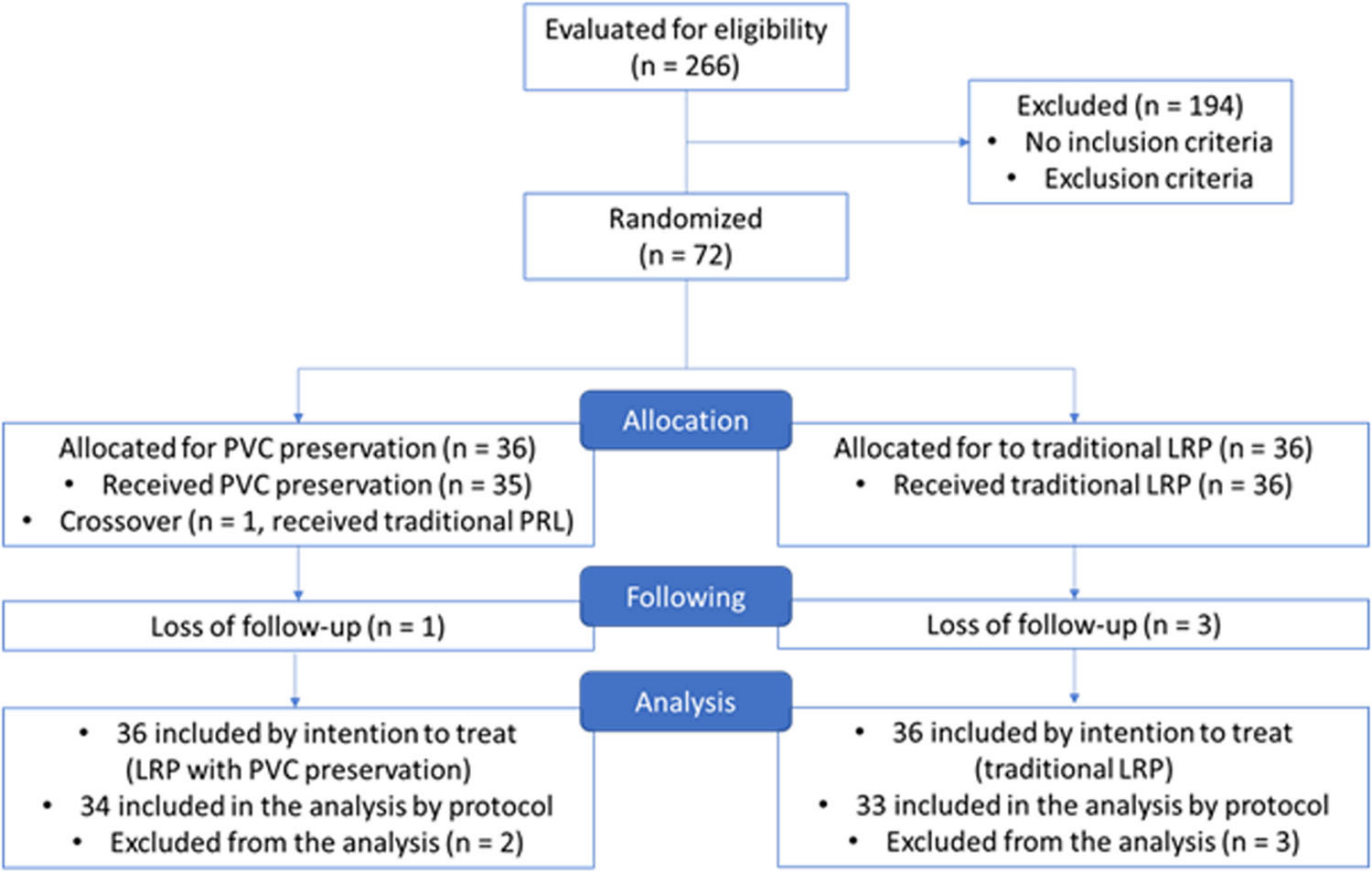
CONSORT model. [Color figure can be viewed at wileyonlinelibrary.com]

Between April 2018 and September 2021, 72 patients undergoing LRP were included in the study. Four patients were excluded from the study due to loss of postoperative follow‐up, and one patient whose PVC preservation procedure was not completed due to technical difficulty. Figure 1, the CONSORT model [9], shows the final number of patients per group. The clinical and demographic data and preoperative tumor staging were homogeneous between the groups, as shown in Supporting Information S1.

### 3.1 Perioperative Results

The perioperative results are shown in Table 1. We observed a significant difference in operative time between the groups, with the preservation group having an average time of 109 min (± 21.47) and the classic technique group having 75 min (± 22.53) of surgery (*p* = 0.000). Blood loss was another variable with a statistical difference Figure 1 between the groups, and the new technique showed an average of 365.59 ml versus 247.27 ml for the control technique (*p* = 0.010).

**Table 1:** Perioperative results.

	Group 1	Group 2	*p* value
Preservation of nerve bundles			
No preservation	11.8% (4)	9.1% (3)	0.549*
Unilateral	52.9% (18)	42.4% (14)	
Bilateral	35.3% (12)	48.5% (16)	
Complications			
No	88.2% (30)	100.0% (33)	0.356**
Yes	11.8% (4)	0.0% (0)	
Surgery time (minutes)	108.94 (±21.47)	75.55 (±22.53)	0.000***
Blood loss (ml)	365.59 (±218.99)	247.27 (±159.26)	0.024****
Length of stay (days)	1.06 (±0.23)	1.09 (±0.24)	0.624***
Catheter time (days)	7 (±0)	7.42 (±1.69)	0.148****

Four operative complications were detected in the PVC preservation group: one was attributed to the technical difficulty in making the vesicourethral anastomosis, another to bladder injury resulting in a wide bladder neck, and the others related to accidental damage to the PVC with its partial preservation. These complications occurred at the beginning of the series but did not lead to further significant adverse outcomes.

The overall postoperative complication rate was 20.6% in the PVC preservation group and 9.1% in the control group, with no statistical difference. The most frequent complication was urinary retention, seen in three patients with PVC preservation and one with the standard technique. Two Clavien grade III complications occurred only in the preservation group: one bladder neck stenosis treated endoscopically and one laparoscopic reoperation for suspected obstructive acute abdomen (Supporting Information S1).

### 3.2 Functional Results

**Figure 2:**
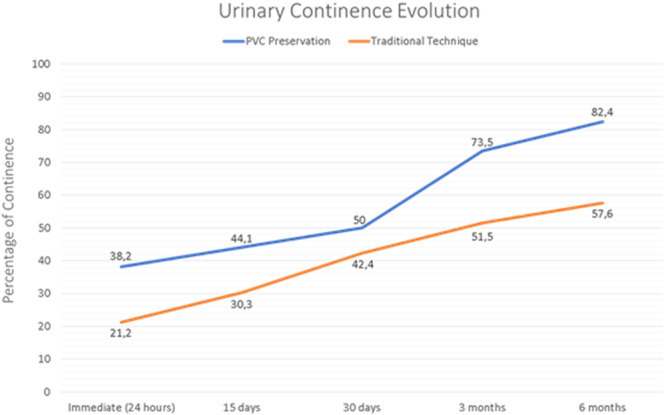
Continence curves throughout the follow‐up period. [Color figure can be viewed at wileyonlinelibrary.com]

The continence curves throughout the follow‐up period are shown in Figure 2. The PVC preservation group remained above the control group and achieved a significant difference at the 6‐month follow‐up. (82.4% vs. 57.6%; *p* = 0.027).

After excluding patients with preoperative erectile dysfunction (IIEF < 17), we analyzed the sexual function of 23 patients with PVC preservation and 21 patients in the traditional technique group. We considered an IIEF higher or equal to 17 as sexual function recovery.

We did not find any statistically significant difference in sexual recovery between groups. The three and 6‐month results were 0 versus 7,4% and 13% versus 23,8% for the PVC preservation technique versus the traditional technique.

### 3.3 Oncological Results

The oncological results were evaluated through the presence of positive margins and the occurrence of biochemical recurrence. We observed a similar rate of positive margins between the groups at 6‐month follow‐up (20.5% vs. 24.2%; *p* = 670). Regarding the location of the margins, group 2 had a predisposition for lateral margins, while the bladder apex and neck regions were the most affected in the PVC preservation group. The biochemical recurrence rate after 6 months of surgery was approximately 10% for both groups (8.8% vs 12.1%; *p* = 0.709).

## 4 Discussion

The evolution and development of new surgical techniques for PC treatment have been frequent in recent years. The advent of the robot as an aid and facilitator of laparoscopy has allowed the emergence of surgical approaches that better preserve the periprostatic anatomy. The best early urinary continence results have been published in preservative techniques that maintain the anterior compartment of the prostate. Asimakopoulos et al. reported in 2010 that 80% immediate continence with preservation of the PVC [3]. In 2013, the Retzius‐sparing technique was studied by Galfano et al., showing a 92% immediate continence rate [10].

These preservations are usually technically challenging, and robotics is suggested as a fundamental piece to perform some of these procedures. However, in 2018, we published the possibility of preserving the PVC by pure laparoscopic approach [4]. The present study is the first randomized trial investigating the PVC sparing technique in LRP and its repercussions on the recovery of urinary continence.

The incidence of postoperative complications with the new technique was 20.6% versus 9.4% for the conventional technique. This rate is similar to a retrospective study that evaluated complications in open and LRP. They analyzed 202 patients undergoing laparoscopic treatment, detecting complications in 19.3% [11].

Analysis of operative data revealed a significant difference in operative time between groups. The mean operative time in the LRP group with PVC preservation was 108.94 min (± 21.47), compared to 75.55 min (± 22.53) in the control group. However, in the initial experience with PVC preservation using robotic surgery, the mean operative time was reported as 132 min (range: 70–180 min) [3], which is slightly longer than the time observed in our laparoscopic adaptation. Furthermore, a systematic review comparing different radical prostatectomy techniques showed mean operative times of 236.54 min (range: 144–400) for laparoscopic surgery, 179.03 min (105–253) for open surgery, and 187.91 min (137–330) for robotic surgery [12].

A higher estimated blood loss was observed in the LRP group with PVC preservation, with a mean of 365.59 ml (± 218.99), compared to 247.27 ml (± 159.26) in the conventional LRP group (*p* = 0.024). However, this increase remains within the expected range for LRP. Notably, in a retrospective study evaluating 757 patients, the subgroup who underwent the transperitoneal approach had a mean estimated blood loss of 308 ml [13], a value that falls between those observed in our two groups.

In this study, continence was defined as the complete absence of urinary leakage and the use of no pads. This strict definition aimed to reduce subjectivity and ensure objective, reproducible comparisons between groups. Given the lack of a universally accepted standard, definitions of early continence recovery vary considerably in the literature. While some authors consider a 3‐month follow‐up a short‐term endpoint after the Retzius‐sparing technique [14], others define early recovery as continence achieved within the first few weeks or within 30 days postoperatively [15].

The 6‐month follow‐up was selected as a relevant endpoint for early functional recovery, as it represents a period of clinical significance and aligns with the timeframe of documented benefits from other preservation techniques. Immediate continence was assessed 24 h after catheter removal. Although the PVC preservation group showed better immediate continence, the difference was not statistically significant (*p* = 0.249). Further evaluations were conducted at 15 days, and at 1, 3, and 6 months. The PVC group showed continuous improvement, reaching 73.5% continence at 3 months and 82.4% at 6 months, compared to 57.6% in the standard group (*p* = 0.032). In contrast, the traditional laparoscopic group reached 51.5% at 3 months, with minimal additional improvement at 6 months.

In this study, we demonstrated a LRP technique capable of replicating the early urinary continence outcomes observed with the most promising robotic approaches, such as the Retzius‐sparing robot‐assisted radical prostatectomy (RS‐RARP). Our continence recovery curve closely resembled those reported with these robotic techniques, particularly at the 3‐ and 6‐month follow‐ups.

A randomized trial published by Dalela et al. in 2017 compared continence outcomes between an anterior approach (Menon's technique) and a posterior approach (Bocciardi's Retzius‐sparing technique). The immediate continence rate, measured 1 week after catheter removal, was 45% for the posterior approach versus 20% for the anterior approach, favoring the Retzius‐sparing technique [14]. When compared to our immediate continence results (38.3% vs. 25%), a similar pattern was observed.

The same cohort was further analyzed by Menon et al. at 6 months of follow‐up. Continence rates, defined as complete absence of urinary leakage at 3 and 6 months, were 75.7% and 91.7% for the posterior approach. These findings are comparable to those observed with our LRP technique involving preservation of the PVC, which demonstrated continence rates of 73.5% and 82.7% at 3 and 6 months, respectively [2].

Further supporting our findings, a 2020 Cochrane systematic review concluded that Retzius‐sparing RARP likely improves urinary continence recovery up to 6 months postoperatively, with moderate‐certainty evidence indicating faster recovery within 1 week of catheter removal and at 3 months after surgery [16].

Although data on erectile function were collected, the relatively short follow‐up period of 6 months, combined with a loss to follow‐up of approximately 30%—all of whom had pre‐existing erectile dysfunction—limited the statistical representativeness of our sample. However, we believe that the ongoing follow‐up at 12 and 24 months for the assessment of continence and sexual function may help elucidate the long‐term outcomes.

While the Retzius‐sparing technique is acknowledged to improve early urinary continence recovery, it remains technically demanding when performed laparoscopically, thereby limiting its widespread adoption outside high‐volume robotic centers. In this context, our proposed technique gains particular relevance, as it combines technical feasibility with the ability to maintain comparable functional outcomes. This makes it a practical and valuable alternative, especially in settings where robotic platforms are not available.

Despite the global expansion of robotic surgery, access to this technology remains limited in many developing countries. In Brazil, robotic systems are mostly concentrated in large urban centers and private hospitals, making them inaccessible to the majority of the population. A cost‐effectiveness analysis conducted by a major Brazilian oncologic center showed that robot‐assisted radical prostatectomy (RARP) doubles the cost of surgery compared to the open approach, representing an additional expense of approximately USD 4000 per procedure [17].

Globally, over 80% of the world's population resides in low‐ and middle‐income countries (LMICs), where healthcare budgets are constrained and high‐cost technologies like robotics are rarely available [18]. Given this context, LRP remains a critical and viable minimally invasive alternative, offering oncologic and functional outcomes comparable to robotic techniques, when performed by experienced surgeons.

Therefore, expanding laparoscopic surgical training and ensuring the availability of laparoscopic equipment are essential steps toward equitable prostate cancer care in resource‐limited settings. To facilitate the adoption of this technique, it is crucial to establish a structured step‐by‐step approach and provide dedicated mentorship, thereby supporting surgeons in overcoming the learning curve and achieving safe and reproducible outcomes. Promoting LRP can bridge the technological gap, ensuring high‐quality treatment for patients regardless of geographic or economic barriers.

## 5 Conclusions

In conclusion, we provide the first evidence that PVC preservation during radical prostatectomy enhances urinary continence recovery. This technique, feasible with standard laparoscopic equipment, offers a valuable and accessible option for surgical centers worldwide.

## Ethics Statement

The clinical trial was conducted after approval by the Research Ethics Committee of the Centro Universitário de João Pessoa under protocol #2.785.823.

## Consent

The participants signed the Informed Consent and Informed Consent Forms before the surgical procedures.

## Conflicts of Interest

The authors declare that they have no known competing financial interests or personal relationships that could have appeared to influence the work reported in this article.

## Supporting information


**Supplementary Material:** Preoperative clinical, demographic and pathological characteristics. **Supplementary Table 2:** Urinary continence results. **Supplementary Table 3:** Erectile function recovery results. **Supplementary Material 4:** Results of surgical margins and biochemical recurrence. **Tabela 7:** Incidência das complicações pós‐operatórias e suas respectivas classificações de gravidade (Clavien).

## Data Availability

The data that supports the findings of this study are available in the supporting information of this article.
