# Supplementary material for: The Pubovesical Complex–Sparing Laparoscopic Radical Prostatectomy Improves Early Urinary Continence Without Compromising Oncologic Safety: A Prospective, Randomized, and Double‐Blinded Clinical Trial
Source: Prostate. 2025 Dec 7;86(4):475–80. doi: 10.1002/pros.70106 (PMC12842851; doi:10.1002/pros.70106)
Supplement: Supplementary file 1 — Supplementary Material: Preoperative clinical, demographic and pathological characteristics. Supplementary Table 2: Urinary continence results. Supplementary Table 3: Erectile function recovery results. Supplementary Material 4: Results of surgical margins and biochemical recurrence. Tabela 7: Incidência das complicações pós‐operatórias e suas respectivas classificações de gravidade (Clavien). [file PROS-86-475-s001.docx]

|  | Group 1  (PVC) | Group 2  (Traditional) | P value |
| --- | --- | --- | --- |
| Age | 61.41 (±5.63) | 64.06 (±5.19) | 0.051*** |
| PSA (ng/dl) | 8.00 (±4.00) | 8.15 (±3.86) | 0.875*** |
| BMI (kg/m²) | 27.21 (±3.54) | 26.61(±3.25) | 0.474*** |
| IPSS | 11.85 (±8.22) | 11.03 (±7.83) | 0.679*** |
| IIEF-5 | 17.65 (±7.45) | 17.72 (±6.40) | 0.967*** |
| Prostatic volume (ml) | 43.15 (±17.88) | 42.56 (±17.96) | 0.895*** |
| Comorbidities |  |  |  |
| No | 50% (17) | 45.5% (15) | 0.710* |
| Yes | 50% (17) | 54.5% (18) |  |
| Clinical staging |  |  |  |
| T1 | 52.9% (18) | 45.5% (15) | 0.540* |
| T2 | 47.1% (16) | 54.5% (18) |  |
| ISUP | 2.06 (0.64) | 2.09 (0.67) | 0.844*** |
| 1 Gleason 6 (3+3) | 17.6% (6) | 18.2% (6) | 0.928* |
| 2 Gleason 7 (3+4) | 58.8% (20) | 54.5% (18) |  |
| 3 Gleason 7 (4+3) | 23.5% (8) | 27.3% (9) |  |
| Commitment side |  |  |  |
| Left | 26.5% (9) | 15.2% (5) | 0.504* |
| Right | 23.5% (8) | 24.2% (8) |  |
| Bilateral | 50% (17) | 60.6% (20) |  |

**Supplementary Material.** Preoperative clinical, demographic and pathological characteristics.

**Supplementary Table 2.** Urinary continence results.

|  | Group 1 | Group 2 | P value |
| --- | --- | --- | --- |
| Immediate urinary continence |  |  |  |
| Continent | 38.2% (13) | 21.2% (7) | 0.128* |
| Incontinent | 61.8% (21) | 78.8% (26) |  |
| Urinary Continence (15 days) |  |  |  |
| Continent | 44.1% (15) | 30.3% (10) | 0.242* |
| Incontinent | 55.9% (19) | 69.7% (23) |  |
| Urinary Continence (30 days) |  |  |  |
| Continent | 50.0% (17) | 42.4% (14) | 0.534* |
| Incontinent | 50.0% (17) | 57.6% (19) |  |
| Urinary Continence (3 months) |  |  |  |
| Continent | 73.5% (25) | 51.5% (17) | 0.063* |
| Incontinent | 26.5% (9) | 48.5% (16) |  |
| Urinary Continence (6 months) |  |  |  |
| Continent | 82.4% (28) | 57.6% (19) | **0.027*** |
| Incontinent | 17.6% (6) | 42.4% (14) |  |

**Supplementary Table 3.** Erectile function recovery results.

|  | Group 1 | Group 2 | P value |
| --- | --- | --- | --- |
| IIEF (3 months) |  |  |  |
| No | 100% (23) | 92.6% (19) | 0.222** |
| Yes | 0% (0) | 7.4% (2) |  |
| IIEF (6 months) |  |  |  |
| No | 87% (20) | 76.2% (16) | 0.448** |
| Yes | 13% (3) | 23.8% (5) |  |

**Supplementary Material 4.** Results of surgical margins and biochemical recurrence.

|  | Group 1 | Group 2 | P value |
| --- | --- | --- | --- |
| Margins |  |  |  |
| Negative | 79.5% (27) | 75.8% (25) | 0.670* |
| Positive | 20.5% (7) | 24.2% (8) |  |
| Vesical | 5.9% (2) | 6.1% (2) |  |
| Apical | 5.9% (2) | 3% (1) |  |
| Lateral | 2.9% (1) | 12.1% (4) |  |
| Anterior | 2.9% (1) | 0 |  |
| Posterior | 2.9% (1) | 3% (1) |  |
| Biochemical recurrence |  |  |  |
| 3 months |  |  |  |
| No | 94.1% (32) | 93.9% (31) | 1.000* |
| Yes | 5.9% (2) | 6.1% (2) |  |
| 6 months |  |  |  |
| No | 91.2% (31) | 87.9% (29) | 0.709** |
| Yes | 8.8% (3) | 12.1% (4) |  |

Tabela 7. Incidência das complicações pós-operatórias e suas respectivas classificações de gravidade (Clavien).

|  | *Grupo 1* | *Grupo 2* | *Valor de p* |
| --- | --- | --- | --- |
| Complicações |  |  |  |
| Sem complicações | 79,4% (27) | 90,9% (30) | 0,305** |
| Complicações | 20,6% (7) | 9,1% (3) |  |
| Clavien 1 | 8,8% (3) | 6,0% (2) |  |
| Clavien 2 | 5,9% (2) | 3,1% (1) |  |
| Clavien 3a | 2,9% (1) | 0 |  |
| Clavien 3b | 2,9% (1) | 0 |  |
